# “COVID affected us all:” the birth and postnatal health experiences of resettled Syrian refugee women during COVID-19 in Canada

**DOI:** 10.1186/s12978-021-01309-2

**Published:** 2021-12-24

**Authors:** Emma Stirling Cameron, Howard Ramos, Megan Aston, Marwa Kuri, Lois Jackson

**Affiliations:** 1 School of Health and Human Performance, Dalhousie University, Halifax, NS Canada; 2 Department of Sociology, University of Western Ontario, London, ON Canada; 3 School of Nursing, Dalhousie University, Halifax, NS Canada; 4 School of Social Work, Dalhousie University, Halifax, NS Canada

**Keywords:** COVID-19, Postnatal healthcare, Refugees, Women, Social support

## Abstract

**Background:**

Prior to COVID-19, postnatal resettled refugee women in Canada reported barriers to healthcare and low levels of social support, contributing to maternal health morbidities. The COVID-19 pandemic appears to be further exacerbating health inequities for marginalized populations. The experiences of resettled refugee women are not fully known.

**Aim:**

To understand Syrian refugee women’s experiences accessing postnatal healthcare services and supports during the COVID-19 pandemic.

**Methods:**

Semi-structured, virtual interviews were conducted with eight resettled Syrian refugee women living in Nova Scotia (Canada) who were postnatal between March and August 2020. Data analysis was informed by constructivist grounded theory.

**Findings:**

Three themes emerged: “the impacts of COVID-19 on postnatal healthcare;” “loss of informal support;” and “grief and anxiety.” Women experienced difficult healthcare interactions, including socially and physically isolated deliveries, challenges accessing in-person interpreters, and cancelled or unavailable in-home services (e.g., public health nurse and doula visits). Increased childcare responsibilities and limited informal supports due to pandemic restrictions left women feeling overwhelmed and exhausted. Stay-at-home orders resulted in some women reporting feelings of isolation and loss, as they were unable to share in person postnatal moments with friends and family, ultimately impacting their mental wellness.

**Conclusions:**

COVID-19 and associated public health restrictions had significant impacts on postnatal Syrian refugee women. Data presented in this study demonstrated the ways in which the pandemic environment and related restrictions amplified pre-existing barriers to care and postnatal health inequalities for resettled refugee women—particularly a lack of postnatal informal supports and systemic barriers to care.

## Introduction

The postpartum period, or the first 12 months after birth, is often a complex, yet exciting time for new parents [1, 2]. It is a time of rapid change when mothers often require timely access to formal reproductive health services and informal supports to facilitate a positive transition into parenthood and support their health and the health of their child(ren) [3, 4]. Access to formal health services provided by obstetricians, primary care providers, doulas, and midwives help women address potential health concerns, including mastitis, cesarian site pain/infection, heavy bleeding, and postpartum depression [1, 2]. The availability of informal supports from partners, family, or friends aids new mothers in balancing the competing demands of early parenthood, supports women’s physical recovery, and emotional wellbeing [5–7]. Positive socioemotional supports in the postpartum period help to mitigate against depressive symptoms and improve maternal self-efficacy [6, 8].

COVID-19 and associated public health restrictions have had a profound impact on the physical and socioemotional health of mothers and families around the globe [9, 10], including those who have given birth or navigated postnatal services during the pandemic [11, 12]. Physical distancing and changes in health service provision has disrupted healthcare access for peri- and postnatal women, with non-essential appointment cancellations, new personal protective equipment requirements, and in some instances, a transition to virtual care [13, 14]. Labour and delivery experiences have been complicated by restrictions on the presence of support people, with some women having to choose between doulas/midwives and partners or friends [11, 14, 15]. Women have often been required to attend perinatal health appointments alone [11, 14] and were forced to cope with rapid healthcare policy changes, impacting birth plans and postnatal preparation [13].

In addition to disrupted healthcare access, COVID-19 restrictions and physical distancing requirements have socially isolated many postnatal women and limited their access to in-person social and informal supports [16–18]. Informal supports (e.g., meal preparation, childcare, advice sharing) provided by family, friends, or community members contributes to women’s postnatal health, mitigates against postpartum depressive symptoms, and contributes to maternal self-efficacy [6, 8]. Limited in-person social contact during the pandemic has left many new mothers isolated and overburdened [19]. Women across the globe have also reported a decrease in their postnatal mental health. Increased rates of depressive symptoms and anxiety, extreme loneliness, isolation, and exhaustion have been reported [11, 12, 14, 17, 19]. Opportunities to share their infant’s milestones and connect with other parents has largely been restricted to online platforms [19]. Women have further described feeling fearful about exposing their babies to the coronavirus [11, 14].

The COVID-19 pandemic has co-occurred with the largest global migration crisis ever recorded [20]. Over 25 million people are currently living as refugees, most of whom have fled from Syria [21]. A refugee is defined as someone who has been forced to flee their country of origin due to a well-founded fear of persecution, conflict, or violence [22, 23]. Canada remains a leader in refugee resettlement, sponsoring over 50,000 Syrian refugees between 2015 and 2021—many of whom are women of reproductive age [24]. The COVID-19 pandemic has exacerbated pre-existing challenges to accessing healthcare for resettled refugees, which is likely to widen health inequities for this population [25–27].

Prior to the onset of COVID-19, refugee and migrant women were already experiencing inequities when accessing postnatal healthcare [28–30]. Resettled refugee women often encounter barriers to care related to language and cultural differences with providers, lack of interpretation services, and limited access to childcare, which contributed to missed or delayed appointments [31–33]. Moreover, refugee women often reported reduced social networks upon arriving in their resettlement country, resulting in lower levels of available informal support after birth [34]. These challenges have contributed to a greater number of unmet physical and mental health concerns among postnatal resettled refugees [35], and a five-times higher risk of developing postpartum depression, when compared to Canadian-born women [28].

Times of crisis often reinforce and exacerbate existing disparities for marginalized populations [36]. Given the impact COVID restrictions have had on non-refugee mothers, resettled refugee women are at an even greater risk of negative postnatal experiences, as a result of their existing barriers to care [25]. Little research has been conducted to understand immigrant, migrant, or refugee women’s postnatal experiences during COVID-19. The overarching aim of this paper is to present data on the postnatal experiences of resettled Syrian refugee women in the context of COVID-19. More specifically, to elucidate refugee women’s experiences accessing postnatal formal health services and informal social supports during the first six months of the COVID-19 pandemic.

## Methods

This research article reports on analyses conducted on a subset of data collected as part of a larger project which interviewed resettled Syrian refugee women in Nova Scotia about their experiences accessing postnatal healthcare and social support. Eight women from the broader data set experienced part of their postpartum period and/or their delivery during the first six months of the COVID-19 pandemic. Their experiences are reported here. Findings on women’s experiences of postnatal services and supports prior to COVID-19 are reported elsewhere [37].

### Setting

This study was conducted in the Atlantic Canadian province of Nova Scotia in the city of Halifax which entered a state of emergency on March 22, 2020 [38]. To slow the spread of COVID-19, a Health Protection Act Order and public health measures were put into place at this time. These measures included physical distancing requirements, gathering limits, travel restrictions, closure of public parks and trails, and the closure of schools and daycare centres. Related to healthcare, many non-essential appointments were cancelled or rescheduled, restrictions were placed on the presence of support people during childbirth and postnatal appointments (zero to one additional person allowed), visits from public health nurses were virtual and some doula services were cancelled, and many healthcare appointments were delivered via telehealth [39]. Nova Scotia’s first wave lasted through March to late May, with restrictions easing in June 2020 [38].

### Methodology

This qualitative study was conducted using elements of constructivist grounded theory [40]. The purpose of utilizing grounded theory for this research was not to develop a theory, but to gain a conceptual understanding of the experiences of resettled Syrian refugees during the COVID-19 pandemic. The practices of grounded theory, first developed by Glaser and Strauss (1967), aid the researcher in developing a set of concepts that theoretically explain a social phenomenon under study [41]. Grounded theorists propose that theories should be ‘grounded’ in data from the field, through the actions, interactions, and social processes of people. A grounded theory seeks to capture the social process in a social context [42] and is well suited to research examining human behaviour relating to healthcare [43]. The constructivist viewpoint acknowledges that theories are constructed by researchers, who are interpreting the constructions of participants, who have sought to understand and explain their own lived experiences [44]. Through these co-constructions researchers use GT to construct an understanding of the issue under study [37].

Grounded theory research techniques used in this study were largely drawn from Corbin and Strauss’s (2008; 2015) methodologies [44, 45]. They state that quality research can be done “without going on to the final step of theory building” and that grounded theory methods can “be useful to researchers who are interested in thick and rich description, concept analysis or simply pulling out themes.” (pp.x-xi) [44] They use the term grounded theory “in a more generic sense to denote theoretical constructs derived from qualitative analysis of data.” (p.1). Corbin and Strauss (2015) encourage researchers to develop their own “repertoire of strategies” (p.89) to fit their research project. Similarly, Charmaz (2014) states that grounded theory is a “constellation of methods” (p.14). In this study, we implemented grounded theory techniques including inductive line-by-line coding, focus coding, and constant comparisons [40]. Techniques of grounded theory were applied to data collection and analysis to develop a comprehensive understanding of the phenomenon. These methods were chosen to highlight the first voice and share the lived experiences of resettled refugee women.

#### Recruitment and procedure

Ethics approval for this project was received by the Dalhousie University Research Ethics Board (#2019-5016). Women were purposively recruited and eligible to participate if they came to Canada as a refugee, originated from Syria, were living in the city of Halifax, and spoke Arabic. The Syrian population was specifically chosen as they currently represent the largest sub-population of incoming refugees in Canada. Immigrants, asylum-seekers, and women from other countries of origin were not included in this study. This paper presents data related to postpartum experiences during the early months of COVID-19 in Nova Scotia, and to be included in the analyses for this report, women had to have given birth after March 2020, or have a child under the age of 12 months at the time of the interview (i.e., had to be postnatal at some point during the months of the pandemic prior to data collection).

A research assistant who was fluent in Arabic and English was hired to assist with recruitment and language interpretation. The research assistant had an undergraduate degree in a health-related field and had significant research, as well as professional interpretation experience. They were also trained on the study procedures and outcomes and worked closely with the lead author throughout the implementation of the project. All recruitment and data collection materials were drafted and reviewed by two English- and Arabic-speaking interpreters to ensure an accurate translation. Women (both those who had COVID-19 postpartum experiences and those who did not) were purposefully recruited through community centres, health clinics, and private sponsorship organizations. Arabic and English versions of recruitment posters were distributed through the social media, websites, email networks, and/or social media platforms of these recruitment groups, as most in-person spaces and events were closed/cancelled due to the COVID-19 pandemic.

Individual, semi-structured interviews were conducted virtually. Potential participants were instructed to contact the research assistant by phone or email to assess for eligibility. Interested participants selected whether they wanted to complete a telephone interview or a video call (Microsoft Teams), and then scheduled a time for the interview. In advance of the interview, women were emailed a copy of the consent form in both Arabic and English. The primary investigator and an Arabic interpreter were present at each interview. Before beginning the interview, the interpreter verbally reviewed the consent form and verbal consent was recorded. Verbal consent helped to alleviate any burden on participants to print, sign, and scan, or electronically sign and email a consent form. Participants were asked a few demographic questions (e.g., marital status, number of children, years spent in Canada) before conducting the interview. They were also asked if part of their postnatal period (i.e., the first 12 months after birth) was during some months of the COVID-19 pandemic. If yes, they were asked a set of additional interview questions specifically related to COVID-19. The interview guide was comprised of a series of open-ended questions based on a review of the literature and suggestions from community stakeholders. Example COVID-related questions included: How did COVID-19 impact your ability to access healthcare services? Were any services unavailable or only available virtually? How did the pandemic impact your ability to see family, friends, or your community? How did this affect you and your mental health/wellbeing? Interviews took place during the months of August and September 2020. All interviews were audio recorded and women were given a $25 gift card as compensation for their time. Following the completion of the interview, participants were asked if they would like to receive a list of services and supports related to postpartum health, mental health, and COVID-19. If yes, this was mailed or emailed to women in both Arabic and English.

### Analysis

Data analysis was conducted in-line with techniques outlined by Charmaz (2014) and Corbin and Strauss (2008; 2015). Audio recordings were transcribed verbatim and translated into English by the same Arabic-speaking research assistant who was present for all interviews. The translated interviews were read a number of times to familiarize the lead researcher with its content. Line-by-line coding was completed using Atlas.ti (version 8) data analysis software [46]. Each line was assigned a key idea. Key ideas were organized into preliminary categories. Constant comparative analysis was used to identify similarities and differences between and within categories, after which related subcategories were collapsed into themes and subthemes through focused coding. Collaborative team meetings were held throughout the coding process to provide feedback and question the first author on the analysis. The research assistant who interpreted and transcribed all interviews reviewed final themes and associated quotes to ensure the results were grounded in the original data and reflected the experiences of the participants.

## Results

Select details of the socio-demographics of the eight women who had postpartum experiences during the early months of COVID-19 are provided in Table 1. All eight participants were married, spoke Arabic as their preferred language, and originated from Syria. Women had between two and eight children and most arrived in Nova Scotia in 2016. All women arrived through the government-assisted resettlement program.

**Table 1:** Participant demographic characteristics

Demographic characteristics	N = 8
Marital status	
Married	8 (100)
SES	
We do not have enough money for basic necessities	0 (0)
We have enough money for basic necessities, but no extras	8 (100.0)
We have enough money to buy extra things beyond necessities, at least sometimes	0 (0)
Prefer not to answer	0 (0)
Number of children	
1–2	1 (12.5)
3–4	2 (25.0)
5–6	3 (37.5)
7–8	2 (25.0)
Sponsorship type	
Government assisted	8 (100.0)
Private sponsorship	0 (0)
Length of time in Canada	
2 years	1 (12.5)
3 years	0 (0)
4 years	7 (87.5)

Three main themes emerged from the data: the impacts of COVID-19 on postnatal healthcare; loss of informal support; and grief and anxiety caused by the COVID-19 environment.

### Impacts of COVID-19 on postnatal healthcare

Participants conveyed that COVID-19 changed their access to and use of postnatal healthcare services. Limitations on in-hospital support people, childcare restrictions, changes to services delivery, and a move towards virtual care created challenges and opportunities for women’s postnatal care.

#### Isolated birthing experiences

Many participants who delivered during the pandemic discussed the impacts that hospital restrictions and larger public health measures had on their birthing experiences. A culmination of factors, including limited access to childcare, restrictions on support people, and the unavailability of doulas, meant that many women laboured and delivered alone, or with fewer supports than they would have liked.

With the temporary closures of public schools, and daycare centres, families had limited options for childcare for their additional children. Participants also did not have extended family (e.g., mothers, sisters) in Canada and many women did not feel comfortable asking friends to watch their children, given their friends’ own responsibilities and the risk of spreading the virus. Several women in this study gave birth during the early months of COVID-19 with no support people, as their husbands took care of their children while they were in the hospital. Participant 1 described her experience: “*No one could accompany me to the hospital because of COVID. I was alone. My husband drove me to the hospital, but I was all by myself during delivery. My husband helped me carry my things with me to the hospital, but other than that I was all by myself.*” Other participants who delivered without support people had similar feelings, describing their births during COVID as “*hard*,” “*scary*,” and “*lonely*.”

Other women who wished for their friends to accompany them to the hospital discussed having to choose between their partners and friends, as only one support person was allowed to accompany women. Participant 7 said, “*My friend was with me. My husband stayed with the kids at home, and he couldn’t even come because during COVID only one person was allowed to accompany the woman who gives birth. He used to bring us food, give them to my friend outside the hospital, but he never entered.*” Visitation after birth was also limited, which was difficult for several participants who had hoped they could see their families or friends after delivery. Participant 3 described how emotionally challenging and isolating it was to not have her partner visit after her birth: “*The hospital rules are strict during COVID. Visits are forbidden, friends can’t come, and they could not be there to help me. My husband was allowed to visit me twice a day in the hospital. I stayed for two days. I felt really lonely, it was a hard experience.*” Participant 1 also mentioned how difficult it was to be separated from her other children while recovering after birth, stating, “*I didn’t see my children for two days, I wanted them to come to the hospital so I could see them, but it wasn’t possible because of COVID. My little baby girl was crying all the time without me… I just wanted to be discharged from the hospital and go home to them.*” (Participant 1).

In addition to the restrictions on support people, several women who delivered during the first wave were unable to access doula services, as doulas were temporarily suspended with the rise in COVID-19 cases. Doulas were frequently used by Syrian newcomer women prior to the pandemic, and as Participant 7 said, “*Before COVID, many Syrian women were offered doulas by [immigrant support group], but I was not offered one. Because of COVID, they stopped this service.*” Not having access to doula support services left women isolated and exhausted without their assistance. Even after doula services resumed, they were not classified as healthcare staff, and had to accompany women as one of their designated support people, limiting the additional family members or friends who could be with participants while they delivered.

#### Changes to interpretation service provision

All participants spoke Arabic as their preferred language and were often dependent on interpretation services to effectively communicate with healthcare providers. Due to public health and hospital policies at the time of COVID-19, most participants reported that use of in-person interpretation services were largely restricted. Postnatal appointments that would have typically been translated by in-person interpreters were moved to telephone interpretation. Telephone interpretation was preferred over no translation at all, but it was often slow. Participant 7 said, “*Before COVID I had an in-person interpreter. But during COVID, it was hard. The interpretation over the phone was not efficient.*” Several women had to wait for telephone interpreters to become available, with one participant waiting six hours in the emergency department for interpretation services to be provided, delaying her access to urgent care. Most participants outlined their preference for in-person interpretation and described how important it can be for interpreters to read women’s body language and facial expressions. As stated by Participant 3, “*The in-person interpreter feels and understands what I am going through just by seeing me. The phone interpreter on the other hand can’t see me and is waiting for me to talk in order to interpret.*” This participant conveyed the added comfort of having a person be present with her and the ease that brings to the appointment.

Participant 3 described how crucial it was for her to have in-person interpretation, particularly during COVID-19, as her husband and friend were unable to be with her during her cesarian section. Not only did this interpreter ensure that the participant was able to communicate with her healthcare team, but she also acted in lieu of the participant’s support people, providing comfort and support: “*When I entered the surgical room I cried. I was so lonely. I was scared. I needed someone to be with me to ease my stress. So, the interpreter, who I thank from the bottom of my heart, told me not to worry and told me that she will be there for me.*” (Participant 3). This participant conveyed the added benefits of in-person interpretation, which go beyond reading body language, to providing emotional support. Other participants even discussed their interpreters acting as advocates for patient safety.

Several participants indicated that they were not given any interpretation at all and were forced to navigate healthcare interactions in English during COVID-19: “*Sometimes they explained things to me by using signs and I understand a little English but it’s hard to understand medical terms and they didn’t use an interpreter for this.*” (Participant 6). This participant felt particularly frustrated by this experience and did not feel like she fully understood the health information shared by her care providers. Moreover, information shared online about changing public health restrictions and hospital-based restrictions was predominantly provided in English. This language barrier made it difficult for women to keep up with the constantly evolving guidelines and restrictions related to COVID-19 and their understanding of access to postnatal care.

#### Transition to virtual care

The transition to telehealth and virtual care had both positive and negative implications for participants. Primary care services and in-home postnatal supports, such as those provided by doulas and public health nurses, were offered virtually. Telehealth appointments did pose some challenges for interpretation. Women described how complicated it was to connect with their primary care providers and interpreters, over the phone. Some appointments were carried out through back-and-forth telephone calls with a translator, who would relay information to the physician and then separately call back the participant: “*I ask the interpreter and she speaks to the doctor and then they get back to me with the answers. It is complicated, but what could we do?*” (Participant 4). Some participants felt a sense of hopelessness around this form of interpretation, feeling as though they had no alternative ways to communicate with providers. Though it was inconvenient for them, they felt it was the only option considering the restrictions caused by the pandemic.

Participants indicated that in-home services provided by doulas and public health nurses during the postnatal period were cancelled in the first wave. While public health nurses continued to follow-up with women virtually, the hands-on care they had provided pre-COVID-19 was not available. Primary healthcare services also transitioned to be mostly virtual during the first wave of COVID-19, impacting women’s ability to access long-acting contraceptives after birth. Women were only able to access condoms or oral contraceptives through virtual care appointments. Participant 4 described her experience: “*I asked the doctor for an IUD insertion. I was prepared for its insertion, but it didn’t happen. Because of COVID, everything was delayed, and until now I haven’t gotten it.*” Though this woman was able to access alternative contraceptive options in the interim, she was particularly interested in long-acting contraceptive options, for their side-effect profile and heightened effectiveness.

Though virtual care did create challenges for some women, it was actually preferred by others. Telehealth appointments alleviated the need for some women to find childcare and transportation to attend in-person appointments. Participant 1 described her preference for virtual care: “*I found the services I got when I delivered during COVID much better than those with my first-born baby here. I liked the services while delivering and the period after. They kept calling me, checking on me and asking how I was doing.*” Similarly, participant seven said telephone appointments were “*much easier*” than in-person visits.

### Loss of informal support

Stay-at-home orders, isolation requirements, and limitations on in-person gathering limited women’s access to informal support people after birth. This was compounded by the temporary closure of schools and daycare facilities, leaving women to care for their children and newborn without external support people, causing exhaustion, fatigue, and isolation.

#### Missing support people

Gathering limits and physical distancing requirements meant that women had few sources of in-person, informal support during their postpartum period. Several participants described the ways in which their neighbours and friends had supported them through previous, non-COVID births in Canada by providing meals, cleaning, offering emotional support and advice, and caring for their other children. These supports were no longer available to women during the first wave of the pandemic because of restrictions concerning household visitation. As all participants had already been separated from their extended family before COVID-19, as a result of forced migration and resettlement, their sense of loss was compounded by also not being able to see local friends after birth, who had become a kind of chosen family. Participant 7 described how difficult it was to be separated from her family and local friends, comparing her emotions to the experience of drowning: “*It was really hard during COVID. In Syria I had my family… but to give birth here with no one with me?! It was really hard. I needed someone with me, my neighbours, my friends… I felt like I was drowning.*” Several other participants had limited social connections even before COVID-19. For these women, nothing had changed between prior births in Canada and their birth during COVID-19, and as one participant explained, “*Things haven’t changed because even before COVID I didn’t have any friends here.*” (Participant 6). These women were already extremely isolated and lonely, having been separated from their family in the Middle East, and having limited or no support people in Canada. Their pre-COVID postpartum experiences were no different than the isolating periods women endured during the pandemic lockdowns.

#### Childcare burden

COVID-19 restrictions also resulted in the closure of schools and daycares between the months of March and June, 2020 [38]. As a result, all participants indicated that they had between one and seven additional children at home, fulltime after delivery. Participants discussed the stress and exhaustion experienced while juggling homeschooling or childcare for their additional children in addition to caring for their new baby. One participant stated, “*There is a lot of stress. My son doesn’t go to daycare because of COVID, so both my sons are home all the time… It was very stressful at home, especially when I felt depressed, and the kids were there all the time… This was the hardest part.*” (Participant 6). Women discussed how tiring it was to have a newborn, let alone care for one in a context with limited support, and while caring for their additional children. Another participant described the social withdrawal that she felt while postpartum during COVID-19 as a result of the physical exhaustion she was feeling, “*I was so tired and fatigued that I didn’t talk to anyone. I didn’t have the energy for anything.*” (Participant 7).

### Anxiety and grief caused by COVID-19

COVID-19 had significant impacts on women’s mental health. The COVID environment overshadowed the joyous moments of childbirth, with women enduring heightened levels of anxiety triggered by a fear of the virus. Participants further reported feelings of sadness and disappointment as their expectations around their birth and postnatal period were disrupted by COVID-19.

#### Fear of COVID-19

A significant source of anxiety for many participants was the risk of themselves or their family being exposed to COVID-19. Women were particularly concerned for their infant’s health, feeling as though they were particularly vulnerable to the virus: “*I was scared of COVID. I was scared over my children’s health and because I had recently delivered, I was afraid of my last baby’s health.*” (Participant 1). Several participants’ husbands were working in essential services (e.g., food delivery, construction) and continued to work during the first wave. This caused concern, for their husband’s health and that the husband may carry the virus into their home: “*I was really scared. Because he was the only one who went outdoors and was exposed to people so I was afraid that he might get infected with the virus and carry it home.*” (Participant 6). Women were particularly hesitant to visit the hospital, or other healthcare clinics, fearing that it was a hotspot for the virus, “*It was hard because I didn’t know how the situation was in the hospital, I was scared that the hospital might be an epicenter of the pandemic. I was scared that the virus spreads easily in the hospital.*” (Participant 6). Fear of COVID-19 was a severe enough that one participant skipped certain postnatal appointments at the hospital. Others described the sadness that it caused them to have to leave their partners and infants at home and visit the hospital alone:

“*I had to take all the precautions measurements and put on a mask and gloves. It was really hard. To go to the hospital a few days after birth, all by myself and to leave my baby at home because nobody is allowed to be with me was really very hard. I was thinking that even if I die, I will die alone. It saddened me*” (Participant 7).

Several women also spoke of the concerns and anxieties they had for family members living in other countries (e.g., the United Kingdom, Lebanon, Syria), where there were high rates of COVID-19: “*In Syria, where my husband’s family is, the situation is very hard, so many COVID cases.*” (Participant 5). Women also spoke of the fear their older children had around the virus. Participant 8 said that her children were “*terrified of COVID.*” Not only did they have to manage their own anxieties around their virus, but their children’s as well, to ensure their mental health was supported through the pandemic.

#### Broken expectations

Several women described the hopes and anticipations that they had about their postnatal experiences, prior to the onset of the pandemic. Many participants were eagerly anticipating their “*Canadian baby*” and were looking forward to sharing this event with their new, local social network of neighbours and friends. Yet physical distancing and gathering limits meant that there were few opportunities for friends to meet and socialize in person with mothers and their new babies. Participants described feeling saddened that this significant life event could not be celebrated in person: “*We really wanted to have a new baby here and were excited… I had been dreaming of having a big party after my birth here, to invite my friends but suddenly COVID happened, and I had no one to support me.*” (Participant 7). Other participants were looking forward to enjoying more intimate moments with their husbands and children, such as having their children visit in the hospital, which was not possible because of COVID-19, leaving women and their families disappointed: “*My kids at home were so upset that they couldn’t visit me at the hospital and see the baby. They had been so excited during my pregnancy saying that they are waiting to visit me in the hospital after I give birth and to hold the baby, they wanted to bring me flowers but of course because of COVID none of that happened.*” (Participant 6). Most participants felt a sense of loss or grief, as they were not able to share in person the joys of having a new baby with their friends and community members—a key moment of the postpartum experience.

## Discussion

This study has revealed the ways in which COVID-19 has impacted resettled Syrian refugee women’s postnatal experiences of formal health care services and informal supports. Participants described experiences of limited support during birth, poor access to in-person interpretation, cancelled in-person health services, and a transition to virtual care. The COVID-19 environment also impacted resettled refugee women’s mental health during the postnatal period, causing increased feelings of fear, isolation, and disappointment. These findings are consistent with other recent reports on the experiences of non-newcomer women who are postpartum during COVID-19 in Atlantic Canada [14, 16, 19]. The COVID-specific experiences of non-newcomer women parallels the pre-pandemic postpartum experiences of resettled refugee women. Isolated birthing experiences [47–49], reduced access to informal support people [48, 50–52], and high rates of postpartum depressive symptoms [28, 53, 54] were all experienced by resettled refugee women before the pandemic began.

Data presented in this study demonstrated the ways in which the pandemic environment and related restrictions amplified pre-existing barriers to care and postnatal health inequalities for resettled refugee women. Particularly, COVID-19 has limited already fragmented access to interpretation [48–50, 55–57]. During the state of emergency in Nova Scotia, the healthcare system restricted access to interpretation, with a move towards telephone interpretation, which was described as cumbersome and slow to access, and less desirable than having an in-person interpreter. Women noted that the function of interpreters goes beyond being a conduit for effective communication between patients and providers. Our findings our consistent with the literature, which has also shown that in-person interpreters provide emotional support and act as a medical advocate for women, ultimately assisting healthcare providers in administering safe, high-quality care [55, 58]. Other studies have highlighted the maltreatment and trauma that can occur during birth when interpreters or interpretation services are not provided [48, 49, 57]. Failure to provide interpreters leaves resettled refugee women vulnerable to inadequate pain management, violates the principle of informed consent, and can leave women distressed [48, 49]. This must be considered in the context of personnel restrictions in hospital and clinic settings as the pandemic continues.

Like other postnatal women in the pandemic, physical distancing requirements and the general fear and anxiety that accompanied the pandemic impacted some participants’ mental health [12, 19, 59, 60]. Women in our study described the stresses and pressures they faced as a result of decreased informal support, increased childcare and homeschooling demands, and the closures/restrictions of valuable support services (e.g., doulas)—all of which have been reported in other literature with non-newcomer women [10, 14, 19]. These stressors were coupled with feelings of disappointment and loss related to missed or robbed birth- and postnatal-related moments and milestones [14, 16, 19]. Studies with non-newcomer women have also found significant increases in postpartum anxiety [12] and depression symptoms [61] during the pandemic, in addition to a significant increase in healthcare visits for postpartum mental health concerns [62].

Though most postnatal mothers in the context of COVID are experiencing worsened mental health outcomes, resettled refugee women in Canada are significantly more likely to experience postpartum depression symptoms—at a rate three-to-five-times higher the Canadian-born women—in a pre-pandemic context. Previous studies have found correlations between increased depressive symptomology and low levels of social and informal support among refugee women [28, 54]. As a result of stay-at-home orders and physical distancing requirements, women were unable to access any in-person support systems they may have established in Canada, which resulted in high rates of isolation and stress. Though this research study did not formally assess or evaluate mental health concerns among resettled refugee women, it could be posited that limited supports and added pressures of the COVID environment may have heightened the risk for postpartum mental illness among resettled refugee women.

The COVID-19 pandemic has posed ethical challenges regarding the implementation of policies that reduce the spread of the virus but may cause unintentional harm to women and their families [11]. Most notably, COVID-related restrictions around the presence of in-person support people in-hospital and at home (e.g., family, friends, doulas) has had detrimental impacts on maternal mental health during the postpartum period [14, 16, 19]. The unavailability of support people left women overwhelmed, exhausted, in a particularly vulnerable stage of life. Members of the Respectful Maternity Care Charter Global Council have called for innovation and flexible programming and policies to deliver high-quality maternal healthcare in a way that respects the rights of mothers and newborns and is COVID safe [63]. Of particular concern is the denial of critical in-person support people during birth, which for our participants included spouses, friends, doulas, and interpreters. We would encourage policymakers to consider doulas and interpreters as necessary and important care team members, and allow them to be classified as such, rather than having to occupy a designated ‘support person’ space. Hospitals could also help to facilitate virtual family-centred care by offering technological supports (i.e., tablet with video capabilities) to connect women with their families virtually during birth and postnatal appointments [64].

### Limitations

This study is not without limitations. Data included in this study were based on a sub-set of findings from a larger study. Although a number of questions were asked specifically about COVID-19, the focus of the parent study and of the interviews was not focused exclusively on women’s experiences during COVID-19. Additional questions were not asked around COVID experiences, as a large portion of the interview was dedicated to pre-COVID experiences and time did not allow for further probing around COVID-19. As the lead researcher is not fluent in Arabic, an interpreter was present for all interviews. The necessary exchanges between the lead researcher and the interpreter cut into the interview time and slowed down the conversation and limited the lead researcher’s involvement in the interviews. Moreover, all analyses were conducted based on English translations of the interviews. It is possible that sentiments or experiences could not be accurately maintained through the translation. However, the translator worked closely with the first author to review parts of the interviews where the Arabic to English translation was not straight-forward. As data were collected during the COVID-19 pandemic, all interviews had to be completed virtually for the safety of the research team and the participants. It is possible that some women did not engage in the study because they did not have access to a telephone or computer or did not have a private space to complete the interview. Lastly, conclusions about pre- and post-COVID changes should be made with caution, as longitudinal data was not collected and not all participants were able to compare postnatal experiences from before and during the pandemic. Further longitudinal research is needed.

## Conclusions

This research paper is one of the first to report on the experiences of resettled refugee women who were postnatal during the early months of the COVID-19 pandemic in Canada. Public health restrictions had significant implications for Syrian refugee women in this study who were in the postnatal period during COVID-19. Participants encountered systemic barriers to postnatal care that had been amplified due to the pandemic. Women’s mental health was also stretched with the competing challenges of new motherhood, limited support, and fear and exhaustion caused by the pandemic, ultimately leading many to feel isolated, anxious, and depressed. The unique barriers facing resettled refugee women must be considered as public health recommendations and restrictions continue to evolve. As we continue to live with COVID-19 and its evolving variants, an equity-oriented approach must be taken to reduce reproductive health disparities for resettled refugee women.

## Data Availability

Participants in this study did not consent to have their transcripts shared outside of the research team. As such, qualitative data is not available for outside use.

## Abbreviation

GT: Grounded theory

## References

[1] Thompson JF, Roberts CL, Currie M, Ellwood DA (2002). Prevalence and persistence of health problems after childbirth: associations with parity and method of birth. Birth.

[2] Brown S, Lumley J (1998). Maternal health after childbirth: results of an Australian population based survey. Br J Obstet Gynaecol.

[3] Darvill R, Skirton H, Farrand P (2010). Psychological factors that impact on women’s experiences of first-time motherhood: a qualitative study of the transition. Midwifery.

[4] Aston M, Price S, Monaghan J, Sim M, Hunter A, Little V (2018). Navigating and negotiating information and support: experiences of first-time mothers. J Clin Nurs.

[5] Negron R, Martin A, Almog M, Balbierz A, Howell EA (2013). Social support during the postpartum period: mothers’ views on needs, expectations, and mobilization of support. Matern Child Health J.

[6] Leahy-Warren P, Mccarthy G, Corcoran P (2012). First-time mothers: social support, maternal parental self-efficacy and postnatal depression. J Clin Nurs.

[7] Emmanuel E, St John W, Sun J (2012). Relationship between social support and quality of life in childbearing women during the perinatal period. JOGNN - J Obstet Gynecol Neonatal Nurs..

[8] Xie RH, He G, Koszycki D, Walker M, Wen SW (2009). Prenatal social support, postnatal social support, and postpartum depression. Ann Epidemiol.

[9] Connor J, Madhavan S, Mokashi M, Amanuel H, Johnson NR, Pace LE (2020). Health risks and outcomes that disproportionately affect women during the Covid-19 pandemic: a review. Soc Sci Med.

[10] Chandler R, Guillaume D, Parker AG, Mack A, Hamilton J, Dorsey J (2021). The impact of COVID-19 among Black women: evaluating perspectives and sources of information. Ethn Health.

[11] Kotlar B, Gerson E, Petrillo S, Langer A, Tiemeier H (2021). The impact of the COVID-19 pandemic on maternal and perinatal health: a scoping review. Reprod Health.

[12] Hessami K, Romanelli C, Chiurazzi M, Cozzolino M (2020). COVID-19 pandemic and maternal mental health: a systematic review and meta-analysis. J Matern Neonatal Med..

[13] Bradfield Z, Hauck Y, Homer CSE, Sweet L, Wilson AN, Szabo RA (2021). Midwives’ experiences of providing maternity care during the COVID-19 pandemic in Australia. Women Birth.

[14] Dol J, Richardson B, Aston M, McMillan D, Tomblin Murphy G, Campbell-Yeo M. Impact of COVID-19 restrictions on the postpartum experience of women living in Eastern Canada: a mixed method cohort study. medRxiv. 2020;1–13. 10.1111/jnu.12843 36380451

[15] Davis-Floyd R, Gutschow K, Schwartz DA (2020). Pregnancy, birth and the COVID-19 Pandemic in the United States. Med Anthropol Cross Cult Stud Heal Illn.

[16] Joy P, Aston M, Price S, Sim M, Ollivier R, Benoit B (2020). Blessings and curses: exploring the experiences of new mothers during the COVID-19 pandemic. Nurs Reports.

[17] Davenport MH, Meyer S, Meah VL, Strynadka MC, Khurana R (2020). Moms are not ok: COVID-19 and maternal mental health. Front Glob Women’s Heal.

[18] Thapa SB, Mainali A, Schwank SE, Acharya G (2020). Maternal mental health in the time of the COVID-19 pandemic. Acta Obstet Gynecol Scand.

[19] Ollivier R, Aston DM, Price DS, Sim DM, Benoit DB, Joy DP (2021). Mental health & parental concerns during COVID-19: the experiences of new mothers amidst social isolation. Midwifery.

[20] UNHCR. Global Trends: Forced displacement in 2019. 2020. https://www.unhcr.org/globaltrends2018/

[21] UNHCR. Figures at a glance. 2020. http://www.unhcr.org/figures-at-a-glance.html

[22] United Nations General Assembly. Convention relating to the Status of Refugees. 1951 [cited 2019 Aug 29]. https://www.ohchr.org/Documents/ProfessionalInterest/refugees.pdf

[23] UNHCR. What is a refugee? 2018; https://www.unrefugees.org/refugee-facts/what-is-a-refugee/

[24] Government of Canada. Syrian refugees - Monthly IRCC updates. 2020.

[25] Germain S, Yong A (2020). COVID-19 highlighting inequalities in access to healthcare in England: a case study of ethnic minority and migrant women. Fem Leg Stud.

[26] Elisabeth M, Maneesh PS, Michael S (2020). Refugees in Sweden during the Covid-19 pandemic—the need for a new perspective on health and integration. Front Public Health.

[27] Kluge HHP, Jakab Z, Bartovic J, D’Anna V, Severoni S (2020). Refugee and migrant health in the COVID-19 response. Lancet.

[28] Ahmed A, Stewart DE, Teng L, Wahoush O, Gagnon AJ (2008). Experiences of immigrant new mothers with symptoms of depression. Arch Womens Ment Health.

[29] Ganann R, Sword W, Black M, Carpio B (2012). Influence of maternal birthplace on postpartum health and health services use. J Immigr Minor Heal.

[30] Sword W, Watt S, Krueger P (2006). Postpartum health, service needs, and access to care experiences of immigrant and Canadian-born women. Lournal Obstet Gynecol Neonatal Nurs.

[31] Heslehurst N, Brown H, Pemu A, Coleman H, Rankin J (2018). Perinatal health outcomes and care among asylum seekers and refugees: a systematic review of systematic reviews. BMC Med.

[32] Khanlou N, Haque N, Skinner A, Mantini A, Kurtz LC (2017). Scoping review on maternal health among immigrant and refugee women in Canada: prenatal, intrapartum, and postnatal care. J Pregnancy.

[33] Riggs E, Davis E, Gibbs L, Block K, Szwarc J, Casey S (2012). Accessing maternal and child health services in Melbourne, Australia: reflections from refugee families and service providers. BMC Health Serv Res.

[34] Higginbottom GM, Safipour J, Yohani S, O’Brien B, Mumtaz Z, Paton P (2016). An ethnographic investigation of the maternity healthcare experience of immigrants in rural and urban Alberta, Canada. BMC Pregnancy Childbirth.

[35] Gagnon AJ, Dougherty G, Wahoush O, Saucier JF, Dennis CL, Stanger E (2013). International migration to Canada: the post-birth health of mothers and infants by immigration class. Soc Sci Med.

[36] Kantamneni N (2020). The impact of the COVID-19 pandemic on marginalized populations in the United States: a research agenda. J Vocat Behav.

[37] Stirling Cameron E, Aston M, Ramos H, Kuri M, Jackson L (2022). The postnatal experiences of resettled Syrian refugee women: access to healthcare and social support in Nova Scotia Canada. Midwifery..

[38] Government of Nova Scotia. Coronavirus (COVID-19): Alerts, news, and data. 2021. https://novascotia.ca/coronavirus/alerts-notices/

[39] Nova Scotia Health. Nova Scotia Health Visitor Restrictions. 2021.

[40] Charmaz K (2014). Constructing grounded theory.

[41] Corbin J, Strauss A (1990). Grounded theory methodology: procedures, canons, and evaluative criteria. Qual Sociol.

[42] Glaser B, Strauss A (1967). Grouned theory: the discovery of grounded theory. Sociol J Br Sociol Assoc.

[43] Wuest J. Grounded theory: The method. In: Nursing research: A qualitative perspective. 5th ed. Sudbury, MA; 2012. pp. 225–56.

[44] Corbin J, Strauss A (2008). Basics of qualitative research: techniques and procedures for developing grounded theory.

[45] Corbin J, Strauss A. Basics of qualitative research: Techniques and procedures for developing grounded theory. Fourth Edition. Thousand Oaks, CA; 2015.

[46] Muhr T. Atlas.ti: The Qualitative Data Analysis & Research Software. 2017.

[47] Ahmed A, Bowen A, Feng CX (2017). Maternal depression in Syrian refugee women recently moved to Canada: a preliminary study. BMC Pregnancy Childbirth.

[48] Henry J, Beruf C, Fischer T (2020). Access to health care for pregnant Arabic-speaking refugee women and mothers in Germany. Qual Health Res.

[49] Stirling E, Aston M, Ramos H, Kuri M, Jackson L (2021). The postnatal experiences of resettled Syrian refugee women: access to healthcare and social support in Nova Scotia, Canada. Midwifery.

[50] Niner S, Kokanovic R, Cuthbert D (2013). Displaced mothers: birth and resettlement, gratitude and complaint. Med Anthropol Cross Cult Stud Heal Illn.

[51] Stirling Cameron E, Aston M, Ramos H, Kuri M, Jackson L (2021). The postnatal experiences of resettled Syrian refugee women: access to healthcare and social support in Nova Scotia, Canada. Midwifery.

[52] Hrabok M, Watterson R, DeVetten G, Wagner A (2020). Canadian refugee women are at increased risk of postpartum depression: how can we help?. J Obstet Gynaecol Canada.

[53] O’Mahony JM, Donnelly TT, Raffin Bouchal S, Este D (2013). Cultural background and socioeconomic influence of immigrant and refugee women coping with postpartum depression. J Immigr Minor Heal.

[54] Stewart DE, Gagnon A, Saucier JF, Wahoush O, Dougherty G (2008). Postpartum depression symptoms in newcomers. Can J Psychiatry.

[55] Wu MS, Rawal S (2017). “It’s the difference between life and death”: the views of professional medical interpreters on their role in the delivery of safe care to patients with limited English proficiency. PLoS ONE.

[56] Correa-Velez I, Ryan J (2012). Developing a best practice model of refugee maternity care. Women Birth.

[57] Origlia Ikhilor P, Hasenberg G, Kurth E, Asefaw F, Pehlke-Milde J, Cignacco E (2019). Communication barriers in maternity care of allophone migrants: experiences of women, healthcare professionals, and intercultural interpreters. J Adv Nurs.

[58] Yeheskel A, Rawal S (2019). Exploring the ‘patient experience’ of individuals with limited english proficiency: a scoping review. J Immigr Minor Health.

[59] Fallon V, Davies SM, Silverio SA, Jackson L, De Pascalis L, Harrold JA (2021). Psychosocial experiences of postnatal women during the COVID-19 pandemic. A UK-wide study of prevalence rates and risk factors for clinically relevant depression and anxiety. J Psychiatr Res.

[60] Adhanom GT (2020). Addressing mental health needs: an integral part of COVID-19 response. World Psychiatry.

[61] Zanardo V, Manghina V, Giliberti L, Vettore M, Severino L, Straface G (2020). Psychological impact of COVID-19 quarantine measures in northeastern Italy on mothers in the immediate postpartum period. Int J Gynecol Obstet.

[62] Vigod SN, Brown HK, Huang A, Fung K, Barker LC, Hussain-Shamsy N (2021). Postpartum mental illness during the COVID-19 pandemic: a population-based, repeated cross-sectional study. Can Med Assoc J.

[63] Jolivet RR, Warren CE, Sripad P, Ateva E, Gausman J, Mitchell K (2020). Upholding rights under COVID-19: the respectful maternity care charter. Health Hum Rights J..

[64] Hart JL, Turnbull AE, Oppenheim IM, Courtright KR (2020). Family-centered care during the COVID-19 era. J Pain Symptom Manage.

